# An open prospective single cohort multicenter study evaluating the novel, tapered, conical connection implants supporting single crowns in the anterior and premolar maxilla: interim 1-year results

**DOI:** 10.1007/s00784-016-2003-0

**Published:** 2016-11-18

**Authors:** Alexander Fügl, Werner Zechner, Alessandro Pozzi, Guido Heydecke, Christine Mirzakhanian, Nikolaus Behneke, Alexandra Behneke, Russell A. Baer, Robert Nölken, Edward Gottesman, Snjezana Colic

**Affiliations:** 1 Bernhard Gottlieb University Clinic of Dentistry, Sensengasse 2a, 1090 Vienna, Austria; 2 University of Rome Tor Vergata, Rome, Italy; 3 University Medical Center Hamburg-Eppendorf, Hamburg, Germany; 4 University Medical Center of Johannes Gutenberg University Mainz, Mainz, Germany; 5 University Associates in Dentistry, Chicago, USA; 6 Private Practice, Lindau, Germany; 7 Private Practice, New York, USA; 8 University of Belgrade, Belgrade, Serbia

**Keywords:** Conical connection, Platform shifting, Anterior maxilla, Immediate temporization

## Abstract

**Objectives:**

The aim of this multicenter prospective clinical study was to evaluate anodized tapered implants with a conical connection and integrated platform shifting placed in the anterior and premolar maxilla.

**Materials and methods:**

The study enrolled patients requiring single-tooth restorations in healed sites of maxillary anterior and premolar teeth. All implants were immediately temporized. Clinical and radiographic evaluations were conducted at implant insertion, 6 months, and 1 year. Outcome measures included bone remodeling, cumulative survival rate (CSR), success rate, soft-tissue health and esthetics, and patient satisfaction. Bone remodeling and pink esthetic score were analyzed using Wilcoxon signed-rank tests. CSR was calculated using life table analysis. Other soft-tissue outcomes were analyzed using sign tests.

**Results:**

Out of 97 enrolled patients (102 implants), 87 patients (91 implants) completed the 1-year visit. Marginal bone remodeling was −0.85 ± 1.36 mm. After the expected initial bone loss, a mean bone gain of 0.11 ± 1.05 mm was observed between 6 months and 1 year. The CSR was 99.0%, and the cumulative success rate was 97.0%. Partial or full papilla was observed at 30.8% of sites at baseline, 87.2% at 6 months, and 90.5% at 1 year. Soft-tissue response, esthetics, and patient satisfaction all improved during the study period.

**Conclusions:**

Bone gain was observed following the expected initial bone loss, and soft-tissue outcomes improved suggesting favorable tissue response using anodized tapered conical connection implants.

**Clinical relevance:**

Rapid stabilization of bone remodeling and robust papilla regeneration indicate favorable tissue healing promoted by the conical connection, platform-shift design.

**Trial registration:**

*clinicaltrials.gov* NCT02175550

## Introduction

Dental rehabilitation of the anterior and premolar maxilla can be a challenging procedure. Adding to this challenge is the fact that both clinicians and patients have set more stringent benchmarks for success [1]. This higher standard has shifted the research focus toward improving hard- and soft-tissue outcomes [2, 3], the esthetics of the restoration, and patient satisfaction [1, 4]. Because maxillary anterior teeth are the most visible part of dentition, poor esthetic outcomes could increase patient distress and dissatisfaction. The first step toward a successful and esthetic restoration is proper implant placement and using the implant that is most appropriate for the indication.

Implant success relies on minimizing motion during the healing process [5], making primary stability paramount. While several patient characteristics affect primary stability, survival can also be improved by selecting an implant with features designed to optimize primary stability. Tapering can improve stability because it compresses cortical bone in areas with inadequate bone [6]. Platform shifting has been shown to preserve crestal bone levels and improve soft- and hard-tissue response [7–14] by moving the microgap location away from the bone [15] and the stress concentration area away from the bone-implant interface [16]. The implant-abutment interface also affects the outcome of implant treatment. Evidence suggests that conical connections may be superior to non-conical ones because they are more mechanically stable and form a tight connection, which reduces micro-leakage and micro-movements [17], maintains torque once the abutment is tightened, and improves abutment stability [18].

All these implant features were combined to develop a novel implant, the NobelReplace Conical Connection (Nobel Biocare AB, Göteborg, Sweden). The popular NobelReplace anodized tapered implant was redesigned to include an internal conical connection with hexagonal interlocking and a built-in platform shift. This design combined with a dedicated drilling protocol provides sufficient primary stability to support immediate provisionalization in many clinical situations [19–21].

To date, only one prospective and one retrospective study using this implant have been published [22, 23]. In both studies, authors evaluated implant placement for a wide variety of indications. The outcome was measured for all tooth locations; in both healed and fresh extraction sites; with a wide range of insertion torques; and using narrow, normal, and wide platform implants. Follow-up was at 2 and 3 years in the retrospective and prospective studies, respectively [22, 23]. While these studies indicated that the tapered conical connection implant is effective and reliable for immediate loading applications, more clinical data are needed to assess the utility of this implant, especially for applications in the anterior maxilla.

The study presented here is an ongoing 5-year prospective multicenter study evaluating this novel tapered conical connection implant for use as an immediately loaded implant placed in healed sites of maxillary anterior and premolar teeth. The primary objective is to assess marginal bone level changes around this implant. The secondary objectives are to evaluate implant success and survival rates, soft-tissue health and esthetics, and patient quality of life. This study is the first multicenter trial evaluating this implant. It also has more limited indications and a longer follow-up than previous studies. Presented here are the 1-year interim results of this 5-year study.

## Materials and methods

This open, prospective, single cohort, multicenter study included patients needing a single-tooth implant-supported restoration in maxillary anterior and premolar teeth (FDI tooth numbering system: 15–25) (*clinicaltrials.gov* NCT02175550). All patients were treated between March 21, 2011 and July 5, 2013 in one of eight participating centers located in Austria, Germany, Italy, Serbia, and the USA. Participating centers included both private practice clinics and academic hospital-based institutions. Centers were selected based on their prior experience with the implant used in this study and their clinical compliance.

The study was approved by the local ethics committee at each institution and was conducted in compliance with the Declaration of Helsinki and according to the industry regulations (the International Conference for Harmonization Guideline for Good Clinical Practice and ISO14155). All patients provided written informed consent prior to inclusion in the study.

Inclusion criteria required that the patient be ≥18 years of age, in good physical and mental health for the duration of the study, and committed to complete the full 5-year term including adherence to the scheduled clinical and radiographic analyses and maintenance. The tooth at the implant site had to have been extracted or lost ≥2 months prior to the implantation, and the implantation site had to be healthy, i.e., free of ongoing active lesions with no tooth remnants, cysts, granulomas, previous tumors or oral cancer, or undergoing radiation therapy. Full-mouth bleeding on probing and full-mouth plaque index had to score ≤25%. The patient had to have a favorable and stable occlusal relationship. The implant site had to be adjacent to natural roots. The 1-stage procedure with immediate temporization without full occlusal loading had to be indicated for the patient’s condition. Patients were allowed a maximum of two single-unit restorations.

Individuals were excluded from the study if they had acute untreated periodontitis; health conditions preventing surgical treatment; any disorders in the planned implant area, e.g., previous tumors, chronic bone disease (such as rheumatoid disease); infections in tissues adjacent to the planned implantation site; or previous oromaxillofacial radiotherapy. Additional exclusion criteria were use of interfering medication (e.g., steroid therapy, or bisphosphonates), alcohol or drug abuse noted in patient records or medical history, heavy smoking (i.e., >10 cigarettes/day), uncontrolled diabetes, severe bruxism or other destructive habits, and pregnancy or lactation. Secondary exclusion criteria at the time of surgery included insufficient bone volume at the implant site to place a ≥3.5-mm diameter, 8-mm length implant, the need for major bone augmentation at the implantation site, or an insertion torque ≤35 or ≥45 N cm.

All implants were placed by experienced surgeons who received training on the study protocol prior to the start of the trial. The insertion torque had to be between 35 and 45 N cm as measured using a manual torque wrench. Implant stability at implant insertion was tested by tapping or rocking the implant with a hand instrument. Bone quality and quantity were assessed at the time of implant placement according to the Lekholm and Zarb classification [24]. The need for bone or soft-tissue grafting at the time of implant placement was determined on a case-by-case basis. The grafting methods used were left to the surgeon’s discretion.

All implants underwent immediate temporization. A cement- or screw-retained provisional crown was placed on a temporary titanium abutment and functionally loaded within 24 h following surgery. The definitive prosthesis, a cement- or screw-retained NobelProcera crown with a titanium or zirconia abutment was loaded within 6 months after implant placement. Decisions regarding the type of abutment and prosthetic-retention method were left to the surgeons’ discretion to ensure they met the individual patient’s needs.

Marginal bone levels were assessed using intraoral periapical radiographs at implant placement (baseline) and the 6-month and 1-year follow-ups. Radiographic examination was performed using a standardized long-cone parallel technique with a custom-made bite block. Images had to be perpendicular to the implant with a clear thread profile and at least 2 mm of surrounding bone visible. Radiographic images could be collected digitally or conventionally. Double films were collected for all conventional radiographs. To prevent inter-rater variability, all bone-height measurements were analyzed by an independent radiologist (University of Gothenburg, Sweden). The bone level was measured as the distance between the most apical bone level to the implant-abutment junction using Adobe Illustrator. Distance was calibrated to the implant diameter, and measurements were accurate to 0.1 mm. Bone levels were recorded mesially and distally, and the average value was calculated for each implant. Marginal bone remodeling was calculated as the difference between the reading at baseline and follow-up examinations. The differences were calculated for the mesial and distal side independently, and the average value was calculated for each implant.

The cumulative survival rate (CSR) was calculated using life table analysis, and the implant success rate was evaluated according to van Steenberghe criteria [25]. Soft-tissue parameters were evaluated as follows. Bleeding on probing (BOP) and plaque accumulation were assessed at the 6-month and 1-year follow-up visits using a modified sulcus bleeding index and modified plaque index, respectively, according to the classification described by Mombelli and colleagues [26]. Soft-tissue contour adjacent to the implant was assessed at implant insertion and 6-month and 1-year follow-ups using the Jemt papilla index (PI) [27]. Pink esthetic score (PES) was evaluated at the definitive prosthesis placement and the 1-year follow-up using the Fürhauser criteria [28]. All soft-tissue parameters were assessed by an independent evaluator (Vienna Medical University, Austria).

In addition, the oral health-related quality of life assessment was performed at the pretreatment visit, implant placement, and the 6-month and 1-year follow-up visits. The Oral Health Impact Profile (OHIP-14) questionnaire was made available in the respective local languages. It rated the prevalence of patients’ functional limitations; physical pain; psychological discomfort; physical, psychological, and social disability; and handicap on a 0–4 scale, where 0 = never, 1 = hardly ever, 2 = occasionally, 3 = fairly often, and 4 = very often [29].

Sample size was calculated using data from a previous implant study [30]. An *α* of 0.05 and a power of 80% were selected. The target sample size was 80 implants. When accounting for 20% subject withdrawal and an equal number of included patients per clinic, the target enrollment was 96 subjects.

Descriptive statistics were used to present the results. The change in bone remodeling was analyzed using a Wilcoxon signed-rank test. The center effect was evaluated using ANOVA with patients nested by center and calculating the mean squares by center, nesting, and residual. The implant CSR was calculated using a life table analysis. The changes in the PI, BOP, and plaque accumulation were analyzed using the sign test. The change in overall PES was collected for one position per patient and was analyzed with the Wilcoxon signed-rank test. Statistical analyses were performed by an independent statistician using the SPSS software version 21.0 (SPSS Inc., Chicago, IL, USA) and SAS System version 9 (SAS Institute, Cary, NC, USA).

## Results

The patient and implant characteristics are shown in Table 1. In this study, 101 patients were enrolled at one of eight centers. Four patients were excluded after source data verification and data monitoring for not meeting inclusion/exclusion criteria. A total of 97 patients were treated with 102 implants (five patients received two implants). There were six patients who dropped out of the study in the first year. Five patients were removed for lack of compliance, i.e., missed follow-up visits, and one dropped out due to implant failure. Ninety-one patients (93 implants) attended the 1-year visit.

**Table 1:** Main patient and implant characteristics

		Number (%)
Patient characteristics		
Age (years)	Mean Range	41.65 18–79
Gender	Female Male	53 (54.6) 44 (45.4)
Smoking habit	Non-smokers Smokers	81 (83.5) 16 (16.5)
Implant characteristics		
Platform diameter (mm)^a^	3.5 4.3	58 (56.9) 43 (42.1)
Implant length^a^	8 10 11.5 13 16	2 (2.0) 18 (17.6) 18 (17.6) 52 (51.0) 11 (10.8)
Position^a^	Central incisor Lateral incisor Canine First premolar Second premolar	10 (9.8) 17 (16.7) 8 (7.8) 31 (30.4) 35 (34.3)
Bone quality^a^	1 2 3 4	6 (5.9) 47 (46.1) 47 (46.1) 1 (1.0)
Bone quantity^a^	A B C D E	32 (31.4) 64 (62.7) 4 (3.9) 1 (1.0) 0
Tissue augmentation	Bone graft prior to surgery Bone graft during surgery Soft-tissue graft	5 (4.9) 16 (17.2%) 14 (13.7)

^a^Data not reported for one implant

All 102 implants were placed in maxillary anterior and premolar teeth. Among the implants placed, 91 (89.2%) were implanted in healed sites, 10 (9.8%) in sites with at least 8 weeks of post-extractive healing, and 1 (1%) was not reported. Overall insertion torque ranged between 35 and 45 N cm (mean 39.2 ± 4.9 N cm). At the time of implant placement, bone and soft-tissue grafting was performed on 16 (17.2%) and 14 patients (15%), respectively. Either autogenous bone (7 patients) or a combination of BioOss and BioGide (9 patients) was used for bone grafting. Soft-tissue grafting techniques included connective tissue grafts (10 patients), pedicle flaps (5 patients), the envelope technique (4 patients), and roll flaps (1 patient). All implants with a recorded assessment (96.1%) were stable at the time of implant insertion.

Information on abutment types was available for 99 implants: 88 received temporary abutments and 11 received final abutments. Data were not reported for 3 implants. The provisional restorations were cement- (*n* = 41) or screw-retained (*n* = 58). Forty-seven (47.9%) of the definitive prosthetic abutments were zirconia, five (5.1%) were titanium, and 44 (44.9%) were classified as “other,” which included 15° Esthetic Abutments and Snappy Abutments 5.5(Nobel Biocare). Data from three patients (3.06%) were missing. Among the definitive prosthetic crowns, 4 patients (4.1%) received acrylic restorations, 83 (84.7%) received ceramic, and 11 (11.2%) received crowns made with unspecified materials.

Mean marginal bone levels at implant placement (baseline), 6 months, and 1 year were −0.37 ± 0.75 mm (*n* = 94; range, −2.27–1.39 mm), −1.35 ± 1.16 mm (*n* = 90; range, −6.77–0.42 mm), and −1.25 ± 1.15 mm (*n* = 85; range, −7.01–1.16 mm), respectively (Table 2). Marginal bone remodeling at 1 year was −0.85 ± 1.37 mm (*n* = 80; range, −7.13–3.07 mm). After initial bone loss during the 6-month healing period (−0.94 ± 1.32 mm, *n* = 84; range, −6.46–1.80 mm), a mean bone gain of 0.11 ± 1.06 mm (*n* = 83; range, −3.07–5.97) was observed between 6 months and 1 year (Table 3). The relatively high standard deviation could partially be due to the center effect, which was found significant for the 0 to 12 months remodeling results. However, no center effect was detected for the remodeling values from 0 to 6 and 6 to 12 months.

**Table 2:** Marginal bone levels throughout the study period

	Implant insertion		6-month follow-up		1-year follow-up	
Mean (mm)	−0.37		−1.35		−1.25	
SD (mm)	0.75		1.16		1.15	
*n*	94		90		85	
Frequency	*n*	%	*n*	%	*n*	%
1.1–2.0	1	1.1	–	–	1	1.2
0.1–1.0	34	36.2	3	3.3	2	2.4
0	3	3.2	1	1.1	1	1.2
−1.0 to −0.1	39	41.5	42	46.7	39	45.9
−2.0 to −1.1	15	16.0	25	27.8	27	31.8
−3.0 to −2.1	2	2.1	12	13.3	9	10.6
−4.0 to −3.1	–	–	5	5.6	4	4.7
<−4.0	–	–	2	2.2	2	2.4
Total	94	100	90	100	85	100

**Table 3:** Marginal bone level changes throughout the study period

	Insertion to 6 months	Insertion to 1 year	6 months to 1 year
Mean (mm)	−0.94	−0.85	0.11
SD (mm)	1.32	1.37	1.06
*n*	84	80	83
*p*	<0.0001	<0.0001	0.0808

The CSR at 1 year was 99.0% (Table 4). Only one implant failure was reported. The failed implant was a 10-mm length regular platform implant. It was placed using simultaneous bone augmentation and implant placement with an insertion torque of 35 N cm in the upper-left first premolar (FDI 24). The implant had to be removed at 1.5-month post-insertion and prior to definitive prosthesis delivery due to a lack of osseointegration. The patient was a smoker (5 cigarettes per day).

**Table 4:** Life table analysis of implant survival

Time period	Implants placed	Failures	Withdrawn	CSR (%)
Placement to 3 months	102	1	1	99.0
3 to 6 months	94	0	3	99.0
6 months to 1 year	93	0	2	99.0

The cumulative success rate after 1 year was 97.0%. Of the 102 implants placed, two implants were considered unsuccessful according to van Steenberghe criteria [25]. One 13-mm length narrow platform implant was mobile 6 months after implant placement. The implant was placed with an initial torque of 35 N cm at position FDI 25. The implant was not removed, and once the implant was stable 6 months later, a new crown was placed at the site. The other patient had an 11.5-mm narrow platform implant that was mobile 5 months after implant insertion. The implant was placed with an initial torque of 40 N cm at position FDI 14. Definitive prosthesis placement was delayed 4 months until adequate implant stability had been achieved.

**Fig. 1:**
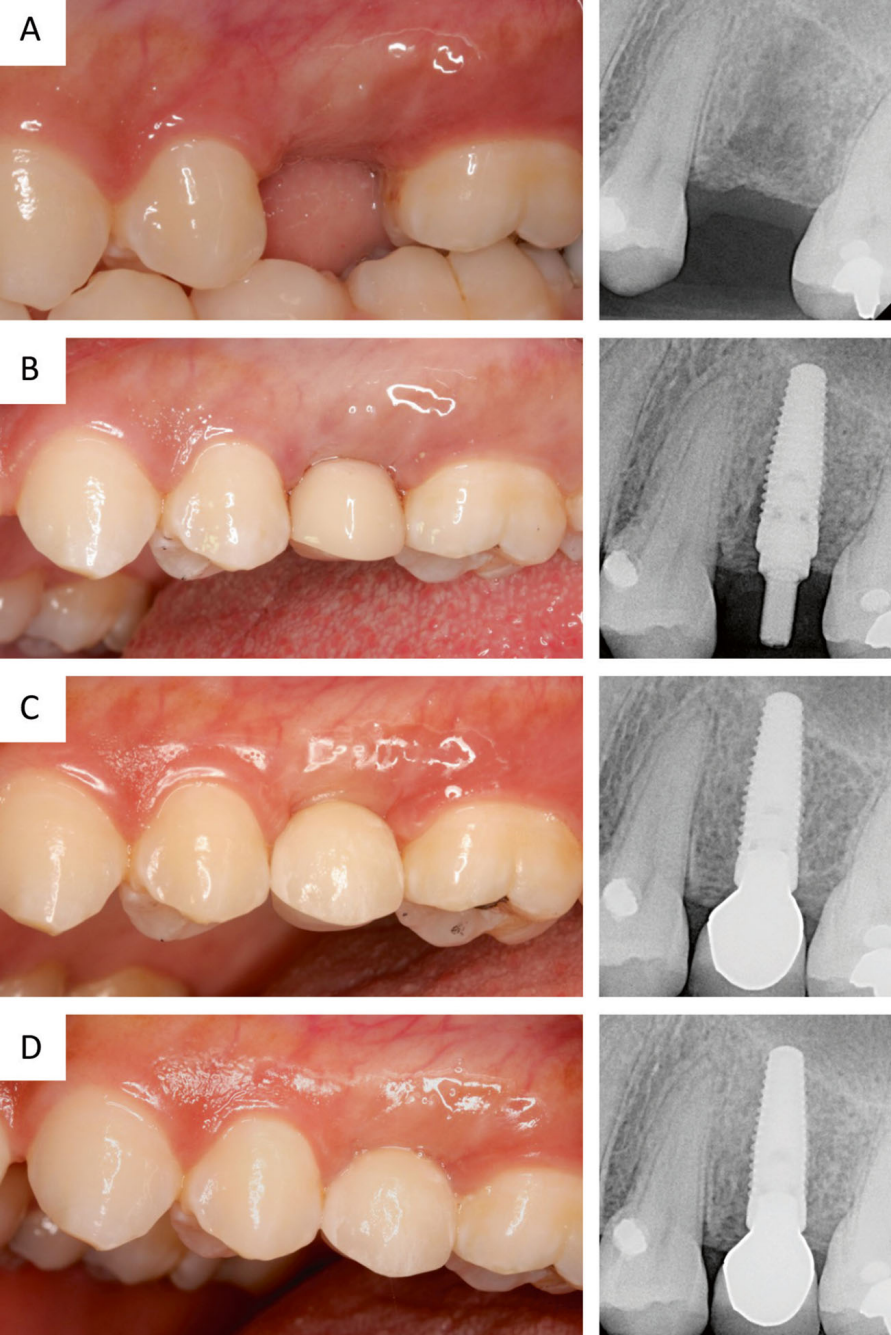
A representative clinical case, position 25. Clinical view and periapical radiograph prior to surgery (**a**), at implant insertion (**b**), at final crown delivery (**c**), and 1 year after implant placement (**d**)

All soft-tissue responses improved over the course of the study. The papilla regeneration was robust. Only 30.8% of implant sites had PI scores of 2 or 3 at placement; however, the number of acceptable PI scores increased to 87.2% at 6 months and 90.5% at 1 year. No visible plaque was detected at 76.6 and 84.3% of implant sites at the 6-month and 1-year visits, respectively. Healthy peri-implant mucosa was observed in most of the patients, with no BOP recorded at 83.3% of implant sites at 6 months and 84.3% of implant sites at the 1-year visit. Furthermore, PES improved significantly (*p* < 0.0001) from a mean score of 8.4 at definitive prosthesis placement to a score of 9.8 at the 1-year follow-up (Table 5). A sample clinical case from the study is shown in Fig. 1.

**Table 5:** PES changes throughout the study period (± standard deviation)

PES variables	Pretreatment (*n* = 84)	Definitive prosthesis (*n* = 84)	1-year follow-up (*n* = 74)
Mesial papilla	0.2 ± 0.4	1.1 ± 0.6	1.5 ± 0.6
Distal papilla	0.1 ± 0.3	0.9 ± 0.5	1.1 ± 0.6
Soft-tissue level	0.9 ± 0.3	1.7 ± 0.5	1.7 ± 0.5
Soft-tissue contour	0.1 ± 0.3	1.1 ± 0.4	1.3 ± 0.5
Alveolar process	0.8 ± 0.6	1.3 ± 0.5	1.3 ± 0.5
Soft-tissue color	0.9 ± 0.4	1.1 ± 0.4	1.3 ± 0.5
Soft-tissue texture	0.9 ± 0.4	1.3 ± 0.5	1.6 ± 0.5
Overall PES	3.8 ± 1.5	8.4 ± 1.9	9.8 ± 2.1

**Fig. 2:**
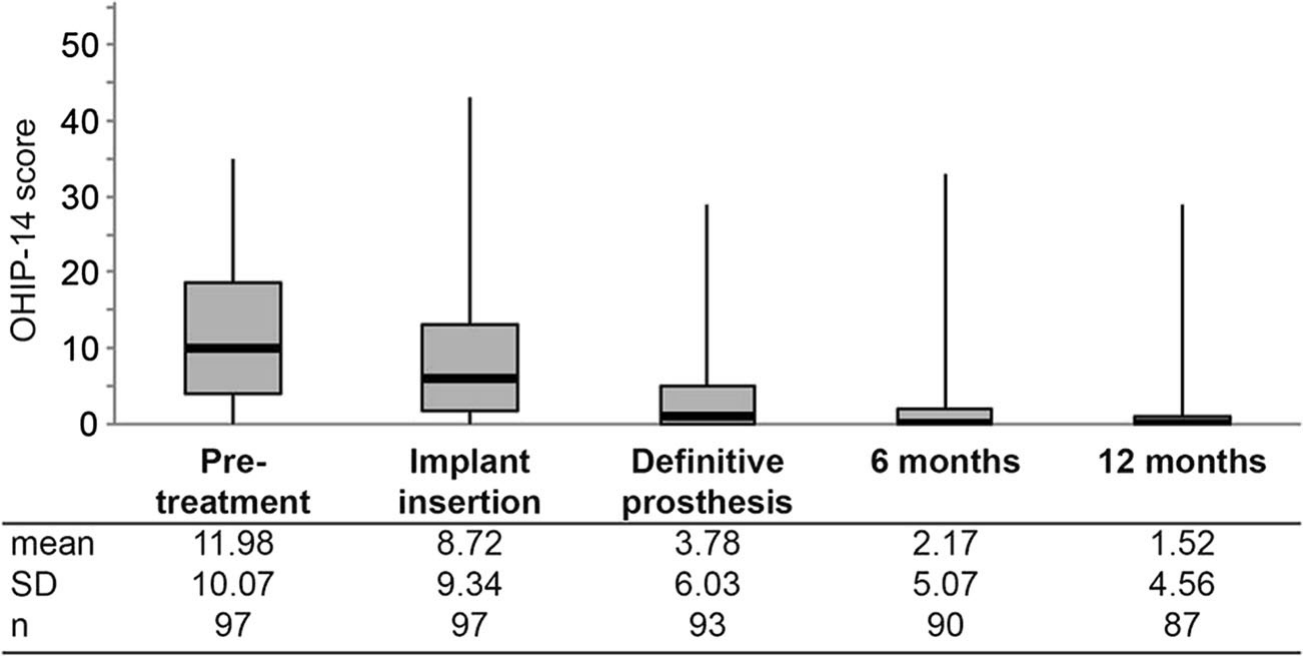
OHIP-14 scores throughout the study. The *black marker lines* indicate the median and the *boxes* signify the first and third quartiles. *Bars* indicate minimum and maximum value. Mean, standard deviation (SD), and sample number per time point (*n*) are listed below the graph

Patient satisfaction improved significantly (*p* < 0.001) based on the mean OHIP-14 score. The mean score was 12 (*n* = 95) at pretreatment, and it decreased to 1.5 (*n* = 87) at the 1-year visit (Fig. 2).

No adverse events other than those described above were reported by the clinicians at the time of data compilation.

## Discussion

The purpose of this ongoing prospective multicenter study is to evaluate the clinical performance of novel tapered conical connection implants placed in healed sites of maxillary anterior and premolar teeth in patients needing single-tooth, implant-supported restorations with immediate temporization. Presented here are the results from the 1-year interim report.

Among various outcome measures, marginal bone remodeling is paramount for implant success. Roos et al. estimated that less than 1-mm marginal bone loss within the first year after implant loading and less than 0.2-mm bone loss each following year is needed to ensure implant success [31]. Marginal bone remodeling at the 1-year follow-up in this study falls within this range (−0.85 ± 1.37 mm). More importantly, after the initial bone loss, a mean bone gain of 0.11 ± 1.06 mm between the 6-month and 1-year follow-ups was observed, indicating healthy hard-tissue response with this implant. However, the marginal bone remodeling values reported here differ from those of previously published studies evaluating the same implant design [22, 23]. In those studies, the mean marginal bone remodeling at 1 year was −0.42 mm. This difference in remodeling is likely due to variations in study protocols. The present study was restricted to implants in maxillary anterior and premolar teeth, whereas the two Pozzi et al. studies did not limit the position of the implant site. In addition, implants in this study were placed in healed sites, whereas Pozzi et al. placed 40–45% of implants in fresh extraction sockets [22, 23]. Studies have shown that post-extraction socket preservation techniques can limit horizontal and vertical ridge alterations, especially within the first year [32]. This limitation could contribute to the low marginal bone remodeling seen in the previous studies. This conclusion was confirmed by the retrospective study, which showed higher bone loss in healed sites compared with extraction sites [22].

The good marginal bone remodeling observed with this implant was reflected in implant survival. Of the 102 implants placed in this study, only one had failed by the 1-year follow-up, resulting in a CSR of 99.0%. In the studies by Pozzi et al., similar outcomes were observed. The prospective and retrospective studies had a 3-year CSR of 98.3% and a 2-year CSR of 99.3%, respectively [22, 23].

Successful implants should also have excellent soft-tissue outcomes. Soft-tissue recovery at the implant site benefits patients’ health, contributes to the esthetic outcome, and strongly affects patient satisfaction [1, 4]. In this study, soft-tissue parameters, including PI, plaque accumulation, BOP, and PES, all improved between implant insertion and the 1-year follow-up. These results are similar to or better than those reported previously for other anodized tapered implants placed in healed sites [33–43]. In this study, 84.3% of implant sites had no BOP at 1 year. This result is comparable to that reported in a study by Kielbassa et al. in which no BOP was observed at 82% of implant sites [34]; however, it is better than those reported by den Hartog et al. [33] and Vasak et al. [42] in which no BOP was observed at 31 and 49.1% of implant sites, respectively. In this study, papilla health improved significantly from 30.8% acceptable scores at placement to 90.5% at the 1-year follow-up. This improvement was comparable to the papilla outcomes reported in previous studies with tapered implants [33, 34, 41]. Importantly, in this study, PES significantly improved, reaching a score of 9.8 at the 1-year follow-up (range 0–14). This result is similar to PES scores reported in den Hartog et al. and Weinländer et al. (7.1 and 10.5, respectively) [33, 43]. Taken together, these data indicate that that this novel tapered conical connection implant supports good soft-tissue outcomes.

While clinical measures are important, the overall goal of implant-supported restorations is to produce functional, esthetic dentition that satisfies the patient. In this study, the OHIP-14 scores showed that patient quality of life significantly increased from pretreatment to the 1-year follow-up. The importance of patient satisfaction has only recently been recognized, and most studies do not evaluate it as an outcome. Therefore, we cannot directly compare the outcomes of this study with those of similar studies to show an improvement in patient quality of life.

As an international, multicenter clinical investigation, the main limitations of the study are related to the inter-center variability and some aspects in the protocol, which were left at the investigators’ discretion. To mitigate the inter-center and inter-operator variability, all participating clinicians received training with the product in the specific indication prior to the initiation of the trial, and bone level measurements were assessed by an independent radiologist. Nevertheless, some center level differences were observed. Another limitation was the variability behind the tooth loss experienced by the treated patients. The conditions leading to a patient’s edentulousness, whether anatomic or pathologic, can affect the complexity of the surgery and the long-term outcomes [44]. Third, while the implant placement protocol was standardized between centers, numerous factors, such as grafting techniques, abutment and prosthetic materials, and prosthetic-retention methods, were left to the surgeons’ discretion. However, to date, there is little high-level evidence supporting the use of any single material or technique, including bone grafting material [45], soft-tissue management technique [46], abutment material [47, 48], crown material [49, 50], or prosthetic-retention method [51]. Given that these protocol variations were not likely to have a large effect on outcomes, decisions about the full treatment plan were made on a case-by-case basis by the clinician. These limitations, when taken together, explain the relatively high standard deviations and ranges observed in this study. This high degree of variability, however, is likely to more closely mirror the outcomes one can expect to see in a clinical practice. The fourth limitation of the study is its dropouts. In total, six patients dropped out by the 1-year visit. The one dropout due to implant failure was accounted for in the CSR; however, an additional five patients dropped out because they did not attend follow-up appointments. While the outcomes of these patients were not measured, it is reasonable to assume that they did not have any complications that might cause a dropout-related change in treatment effect, and the handling of the statistics was treated accordingly [52]. Furthermore, the sample size calculation allows for study dropouts and enables sufficient statistical power to support evaluation of study outcomes.

## Conclusion

Because the anterior and premolar maxilla are a highly visible area, both the clinician’s and the patient’s expectations are high. Within the limitations of the present study, these data suggest that the novel, tapered, conical connection implant produces good clinical results at 1 year. The trend toward bone gain after the initial bone remodeling and improved soft-tissue health suggest favorable tissue response at the implant site and demonstrate that this implant could be a valuable and reliable treatment option for immediate implant loading of single crowns in healed sites of the anterior and premolar maxilla.
